# MicroRNA: A Key Player for the Interplay of Circadian Rhythm Abnormalities, Sleep Disorders and Neurodegenerative Diseases

**DOI:** 10.3390/clockssleep2030022

**Published:** 2020-07-23

**Authors:** Chisato Kinoshita, Yayoi Okamoto, Koji Aoyama, Toshio Nakaki

**Affiliations:** 1 Department of Pharmacology, Teikyo University School of Medicine, 2-11-1 Kaga, Itabashi, Tokyo 173-8605, Japan; ciitaka@med.teikyo-u.ac.jp (C.K.); yayoimya841@med.teikyo-u.ac.jp (Y.O.); kaoyama@med.teikyo-u.ac.jp (K.A.); 2 Teikyo University Support Center for Women Physicians and Researchers, 2-11-1 Kaga, Itabashi, Tokyo 173-8605, Japan; 3 Faculty of Pharma-Science, Teikyo University, 2-11-1 Kaga, Itabashi, Tokyo 173-8605, Japan

**Keywords:** microRNA, circadian rhythm, clock gene, sleep disorder, neurodegenerative disease, biomarker, therapeutic

## Abstract

Circadian rhythms are endogenous 24-h oscillators that regulate the sleep/wake cycles and the timing of biological systems to optimize physiology and behavior for the environmental day/night cycles. The systems are basically generated by transcription–translation feedback loops combined with post-transcriptional and post-translational modification. Recently, evidence is emerging that additional non-coding RNA-based mechanisms are also required to maintain proper clock function. MicroRNA is an especially important factor that plays critical roles in regulating circadian rhythm as well as many other physiological functions. Circadian misalignment not only disturbs the sleep/wake cycle and rhythmic physiological activity but also contributes to the development of various diseases, such as sleep disorders and neurodegenerative diseases. The patient with neurodegenerative diseases often experiences profound disruptions in their circadian rhythms and/or sleep/wake cycles. In addition, a growing body of recent evidence implicates sleep disorders as an early symptom of neurodegenerative diseases, and also suggests that abnormalities in the circadian system lead to the onset and expression of neurodegenerative diseases. The genetic mutations which cause the pathogenesis of familial neurodegenerative diseases have been well studied; however, with the exception of Huntington’s disease, the majority of neurodegenerative diseases are sporadic. Interestingly, the dysfunction of microRNA is increasingly recognized as a cause of sporadic neurodegenerative diseases through the deregulated genes related to the pathogenesis of neurodegenerative disease, some of which are the causative genes of familial neurodegenerative diseases. Here we review the interplay of circadian rhythm disruption, sleep disorders and neurodegenerative disease, and its relation to microRNA, a key regulator of cellular processes.

## 1. Introduction

Most researchers agree that the earth began to form approximately 4.6 billion years ago. Although the primitive Earth apparently had an unstable rotation, the rotation cycle seems to have been fixed at around 24 h by the time the first living organisms appeared. The 24-h rotation produces periodic changes in environmental conditions, such as solar energy day/night lighting cycles. Probably due to the efficient adaptation to the cycle of environmental changes generated by the Earth’s 24-h rotation, living organisms internalize a cell-autonomous clock, the so-called circadian clock. From bacteria to humans, most living beings possess a circadian clock in the body [1,2], and this characteristic of organisms persisted even across explosive evolution and mass extinction events, suggesting that circadian clock systems were internalized in living beings from early in their evolution rather than acquired later as a trait. 

The circadian clock system is technically based on a transcription–translation feedback loop described by the concept of central dogma, which produce rhythmic gene expression, in a process that involves many regulatory steps [3]. The circadian clock is regulated by so-called clock gene(s), and the expression of most of these genes also oscillates in a circadian manner. Indeed, many physiological activities are under the control of circadian regulation through the rhythmic regulation of gene expression. Generally, an abnormality of the circadian clock in itself is not directly lethal to living beings since laboratory animals without clock gene(s) are viable and fertile [4]. However, it is increasingly clear that animals that are deficient in or overexpress clock gene(s) are susceptible to various diseases, leading to fatal results [5]. 

According to the RNA World concept, RNA or RNA-like chemicals carried out most of the information processing and metabolic transformations needed for biology to emerge from chemistry in the early history of life [6]. RNA has several roles, acting as a DNA “photocopier”, a protein building block, a structural component of ribosomes and ribozymes and a regulator of cellular processes. Thus, life may have begun with and evolved along with RNA. If we consider this RNA World concept together with the presumed establishment of the circadian clock in the earliest life forms on Earth, it seems reasonable to assume that the circadian system and RNA regulation evolved together, and that there was some interplay between them. It has long been thought that non-coding RNAs which cannot translate into a protein product are all “junk”, but more recently this assumption has been disproven [7]. Indeed, non-coding RNAs are now a hot topic in research. Many non-coding RNAs are very much functional in biological systems and compensate for their inability to be translated into proteins through alternate mechanisms. MicroRNAs (miRNAs) are a class of non-coding RNAs that function as post-transcriptional regulators. MiRNAs play a role in regulating several factors that are important for biological systems in the body [8]. Recent lines of evidence show that the circadian rhythm of gene expression is regulated by miRNAs, and vice versa [9]. The molecular mechanism of the circadian clock is precisely controlled by post-transcriptional and post-translational regulation, based on a well-organized transcription and translation feedback system.

However, the breakdown of the circadian clock system has been suggested to lead to various diseases in the body. In the case of a breakdown in the central nervous system, this could be a cause of neurological diseases, including sleep disorders and neurodegenerative diseases. Since sleep disruption can be an early symptom in the development of neurodegenerative disease, it may play a role in the development and progression of these diseases. Moreover, miRNAs that are abnormally expressed in the blood, body fluids and/or several tissues are often shared in common between patients with sleep disorders and patients with neurodegenerative diseases [10]. These facts implicate that miRNAs, through their ability to modify the expression of genes related to or causative of diseases, could be both biomarkers of disease pathogenesis and effective therapeutics. 

## 2. Molecular Basis of Circadian Clock System

**Figure 1:**
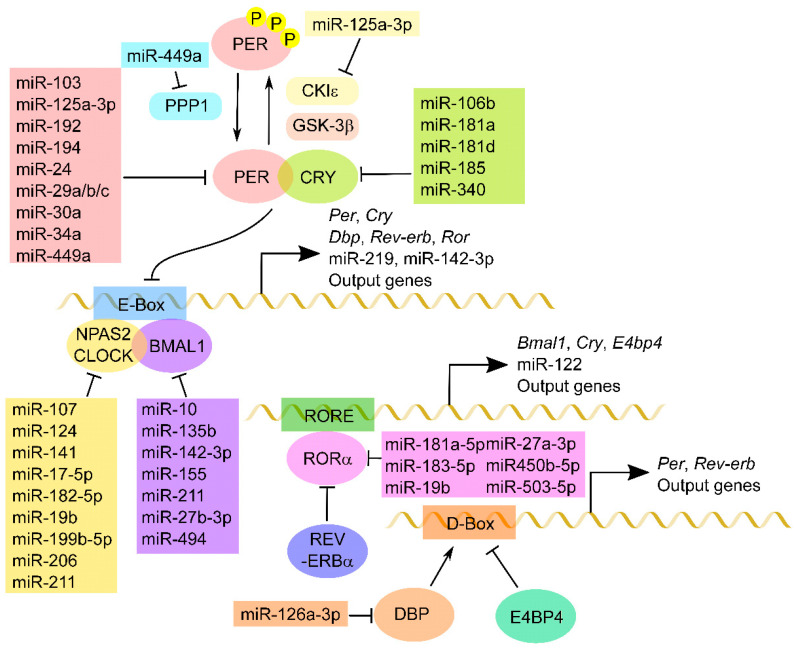
MiRNA regulation of core clock components. A schematic of a molecular circadian system composed of core clock genes is shown. The transcription factors CLOCK:BMAL1 bind to target E-Box and activate the clock genes *Per*, *Cry*, *Dbp*, *Rev-erb* and *Ror*, as well as clock-controlled genes, including miRNAs. After PER and CRY are synthesized in the cytoplasm, these proteins form a complex and inhibit CLOCK:BMAL1-mediated transactivation. PER proteins are phosphorylated by CKIε and/or GSK-3β and dephosphorylated by PPP1 in order to regulate the cellular distribution and/or stability. In turn, RORα activates whereas Rev-erbα reduces the transcription of *Cry*, *Bmal1* and miRNAs and other output genes that have RORE in the region upstream of the promoter. The DBP-dependent transactivation is repressed by competitive binding of E4BP4 to the D-box. Several miRNAs directly down-regulate these core clock components and modulate circadian rhythm.

Living organisms possess circadian systems to adapt to Earth’s 24-h solar energy cycles [11]. The circadian system is based on the molecular mechanisms regulated by several clock genes, such as those encoding transcriptional activators, repressors, and modification enzymes. The classical molecular mechanism of the circadian clock is composed of several feedback loop systems, including a transcriptional and translational step. In mammals, the first loop includes the positive elements CLOCK and BMAL1, as shown in Figure 1, which are members of the basic helix–loop–helix Per–Arnt–Sim transcription factor family. A heterodimer of CLOCK and BMAL1 activates the transcription of target genes containing E-box cis-regulatory enhancer sequences, including the members of the clock gene family, *Period* (*Per1-3*) and *Cryptochrome* (*Cry1, 2*). Negative feedback is achieved by heterodimers of the PER and CRY proteins, which translocate back to the nucleus to repress their own transcription by acting on the CLOCK:BMAL1 complex. CLOCK can be substituted with neuronal PAS protein 2 (NPAS2), a paralog of CLOCK that dimerizes with BMAL1 to form transcriptionally active complexes [12]. NPAS2 can compensate for the loss of CLOCK in peripheral cells as well as in SCN. The second loop includes the retinoic acid-related orphan nuclear receptors (RORs), REV-ERBα and RORα, acting through enhancers of the ROR response element (RORE). CLOCK:BMAL1 heterodimers activate the transcription of *Rev-erbα* and *Rorα*, which subsequently compete to bind ROREs present in the *Bmal1* promoter, although the two proteins have opposite effects on *Bmal1* transcription. The third loop includes basic leucine zipper transcription factors of the proline and acid amino acid-rich subfamily (DBP, TEF and HLF) as positive regulators and E4BP4 as a negative one. These factors act antagonistically on the D-box (the D-site of the albumin promoter) element in their target genes, including *Per1-3* and *Ror α*. These loops interact in an intricate manner to compose the basic architecture of the circadian clock. The autoregulatory feedback loops of the molecular circadian clock take 24 h to complete a cycle. The generation of precise 24-h cycles is governed by post-translational modifications, including phosphorylation, ubiquitination and acetylation. The phosphorylation of PER proteins by casein kinase Iε (CKI ε) and glycogen synthase kinase-3β (GSK-3 β) promotes their nuclear translocation. Further, the phosphorylation of PER at other sites by CKIδ/ε also promotes β-TrCP-dependent PER ubiquitination. CKI-mediated PER phosphorylation is antagonized by phosphoprotein phosphatase1 (PPP1) dephosphorylation. In addition, the phosphorylation of CRY by tyrosine-phosphorylation-regulated kinase 1A (DYRK1A), GSK-3 β and adenosine monophosphate-activated protein kinase (AMPK) leads to its degradation dependent on either F-box and leucine rich repeat protein 21 (FBXL21) or FBXL3-mediated ubiquitination. Interestingly, CLOCK itself has an acetylation activity to BMAL1, which in turn is deacetylated by rhythmic deacetylase Sirtuin1 (SIRT1) (for a more detailed description see [11,13]).

## 3. MiRNA Biosynthesis

MiRNAs are a class of short non-coding RNAs which are approximately 20 nucleotides in length [14]. Their function consists mostly of silencing target expressions by binding to target gene transcripts located mainly at the 3′-untranslated regions (3′-UTR). Most miRNAs are located in intergenic regions or in an antisense orientation to gene regions on the genome. Clustered miRNAs can either be simultaneously transcribed from single polycistronic transcripts containing multiple miRNAs or independently transcribed. In brief, the biogenesis of miRNAs is as follows. First, the primary miRNAs (pri-miRNAs)—which are primary transcripts containing stem-loop structures and are usually thousands of nucleotides in length—are transcribed by a polymerase, in most cases RNA polymerase II. Second, the pri-miRNAs are cleaved by a complex called a microprocessor containing the ribonuclease III Drosha and the RNA-binding protein DGCR8/Pasha, which generate small hairpin-shaped RNAs of approximately 70–100 nucleotides in length, called miRNA precursors (pre-miRNAs). Third, pre-miRNAs exported by exportin-5 in complex with RAN-GTP are processed by a double-stranded ribonuclease III enzyme, termed Dicer, which is complexed with a double-stranded RNA-binding protein. Fourth, the mature miRNA duplexes are loaded onto an Argonaute protein to form an effector complex, called the RNA-induced silencing complex (RISC). Finally, one strand of the miRNA is removed from RISC to generate the mature RISC that induces gene silencing. The post-transcriptional regulation by the RISC complex is mediated by incomplete base-paring of miRNA–mRNA interactions, likely due to the targeting of multiple transcripts, which contributes to the complexity or redundancy of miRNA systems [14]. 

## 4. Circadian Regulation of miRNA

The expressions of the mature or precursor forms of some miRNAs exhibit circadian and/or diurnal rhythms, although the mechanism remains unclear [15]. The circadian rhythmicity of miRNA expression appears to be conserved from plants to mammals [13]. In addition, some of the miRNAs that display circadian rhythm have been reported to contain the circadian cis-elements E-Box and RORE in their upstream regions [9,16,17]. In the mammalian SCN, miR-219 is rhythmically expressed and knockdown within the SCN results in the lengthening of the period of behavioral rhythms [9]. The promoter of miR-219 possesses a non-canonical E-box element and is activated by CLOCK: BMAL1. MiR-122 is also rhythmically expressed, potentially through REV-ERBα acting on two ROREs in its promoter [17]. In addition, the rhythmic expression of miR-142-3p, which is likely driven by a canonical E-box within its promoter, is observed in cultured fibroblasts following serum shock, in immortalized SCN cells and in the murine SCN [16]. In another study, miR-132 was shown to affect light-inducible clock-entrainment in the SCN [9]. There is a CRE promoter in its upstream region that shows light-inducible activation by CREB in a MAPK/ERK-dependent manner [9]. Further, miR-132 and miR-212, which are in the same cluster, modulate the entrainment of the seasonal photo period by regulating the dendritic spine density of SCN neurons acting through the methyl CpG-binding protein rhythm [18]. Dicer has been reported to show a diurnal pattern of expression in various tissues, which may affect the mature miRNA rhythm [19,20]. However, these reports are not sufficient to describe the mechanism of miRNA rhythms, since not all the miRNAs have circadian cis-elements in their promoters and the rhythm generation of mature miRNAs is dependent on the rhythm of both their transactivation and processing. In addition, there is significant discrepancy among the lists of mature miRNAs with circadian expression published by several research groups using high-throughput technologies, including microarray, RNA-sequencing and ChIP-sequencing [17,21,22,23]. Further research will be needed to more fully understand the mechanism of circadian generation and regulation of miRNAs.

## 5. Interplay of Circadian Genes and miRNAs

Several lines of recent evidence show that clock components are also regulated by miRNAs. The Period genes, *Per1*, *Per2* and *Per3*, which are regulated in mammals by common miRNAs, such as miR-24, miR-29 family members (miR-29a, miR-29b and miR-29c), miR-30a, miR-34a-5p, miR-192 and miR-194, are likely to be involved in the timekeeping mechanism in most tissues, given that they are widely expressed in several types of cells or tissues [21,24,25,26,27]. Further, *Per2* is likely to be regulated by miR-449a, which also targeted *PPP1*, a dephosphorylation enzyme of PER2 phosphorylation by CKIε according to the computational analysis of microarray data using the SCN of Clock-mutant mice [30]. In addition, this analysis also showed that both *CKIε* and *Per3* are targets of miR-125a-3p [28]. Moreover, *Per3* is regulated by miR-103 in colorectal cancer cells, and the expression of miR-103 is known to be induced by *Bmal1* in the vascular smooth muscle cells [29,30]. The pre-miRNA constructs of miR-142-3p and miR-494 show circadian rhythm in the serum and reduce *Bmal1* transcription in the SCN cells [16,31]. The rhythmic expression of miR-27b-3p and miR-155 plays a role in regulating the rhythmic expression of *Bmal1* mRNA and protein levels in the mouse liver [32]. It is of interest to note that *Bmal1* inhibits the induction of miR-155 via interfering with the activation of the inflammatory pathway, and miR-155 directly targets *Bmal1* to control circadian inflammatory responses in macrophages [33]. In addition, miR-135b directly targets the BMAL1 3’-UTR and asynchrony between miR-135b and BMAL1 expression impairs the local circadian control in pancreatic cancer cells [34]. MiR-10a contributes to the down-regulation of the expression of *Bmal1*, which is involved in abnormal liver metabolism in cirrhotic liver [35]. Furthermore, miR-211 directly regulates *Bmal1* and *Clock* via distinct mechanisms and contributes to cell survival in Burkitt’s lymphoma cell lines [36]. *Clock* is known to be a target gene of multiple miRNAs, such as miR-107, miR-124, miR-141, miR-17-5p and miR-182, although the circadian rhythmicity of CLOCK is still ambiguous [37,38,39,40,41,42,43,44,45]. The targets of miR-17-5p also include *Npas2*, a paralog of *Clock*, which may play a role in shortening the period [44]. In addition, miR-199b-5p targets *Npas2* to promote the reprogramming of glucose metabolism in hepatocellular carcinoma cells [46]. *Clock* was validated as an mRNA target of miR-206, which may participate in the progression of glioma [47]. Further, *Clock* and *Rorα* can be regulated by miR-19b in the nervous system, and miR-19b might play a role in the development of posttraumatic stress symptoms [48]. *Rorα* was also cooperatively suppressed by miR-503-5p, miR-450b-5p, miR-27a-3p, miR-181a-5p and miR-183-5p in HeLa cells [49]. Pre-miR-185 directly binds to the 3′-UTR of the *Cry1* gene and regulates CRY1 oscillations by reducing CRY1 translation in the NIH3T3 cells [50]. *Cry1* is also a target gene of miR-181a, which plays a role in alleviating the degree of kidney injury through the suppression of the immune-response pathway [51]. *Cry2* is repressed by miR-7-5p, which is transcriptionally activated by the signal transducer and activator of transcription 3 (STAT3) to induce osteoblast differentiation [52]. Both miR-106b-5p and miR-181d play a role in enhancing cancer cell growth by downregulating *Cry2* [53,54]. *Cry2* could also be a target of miR-340, which is involved in the early life programming of anorexia [55]. *Dbp* could be regulated by miR-126a-5p and has a potential for the treatment of hypertension and stroke [56]. Interestingly, a miRNA cluster consisting of miR-96, miR-182 and miR-183 shows diurnal expression, and this cluster is suggested to be involved in circadian rhythm regulation, perhaps by modulating the expression of adenylyl cyclase VI in the retina [57]. Since miRNA regulation could specifically occur in certain tissues, these interplay of circadian genes and miRNAs may also be tissue specific [58]. However, these findings indicate that miRNAs play an important role in mediating between circadian rhythm and physiological function. Recent studies have revealed an obvious disagreement between the number of rhythmic mRNAs and that of proteins [59,60,61]. The number of oscillating proteins is much greater than that of mRNAs, suggesting the presence of a strong network between the circadian system and miRNA regulation.

## 6. Sleep Disorder, Circadian Rhythm Disorder and miRNA Regulation

Sleep is essential for various aspects of brain function, including cognition, concentration, productivity, and performance. Sleep deprivation causes not only several health problems but also serious diseases. Sleep loss changes the cerebral levels of miRNAs, such as miR-125a, miR-132 and let-7 family members [62]. Further, rapid eye movement (REM) sleep deprivation has been shown to considerably affect the hippocampal expressions of miR-132, miR-182, and miR-124 [63]. In addition, chronic short sleep is associated with a marked reduction in the circulating levels of miR-125a, miR-126 and miR-146a [62,64]. MiR-125a, which could target *Per3*, as well as *CKIε*, is preferentially expressed at the end of the active period in rodents and appears to be involved in the long-term regulation of sleep [65]. Further, miR-125a and miR-146a exhibit circadian patterns that decrease and phase shift in aged or diabetic mouse retina in tandem with changes in Dicer expression [19,66]. MiR-132 and miR-182 play an important role in modulating the circadian clock system as described above. These results have implications for the interplay of abnormal expression patterns in circadian miRNAs and several sleep problems.

Sleep disorders are frequent and can have serious consequences for patient health and quality of life. There are various symptoms of sleep disorders, including insomnia (difficulty in falling asleep, sleep fragmentation, early morning awakening), excessive daytime sleepiness, circadian rhythm changes, REM sleep behavior disorder, periodic leg movements in sleep, restless leg syndrome, central or obstructive sleep apnea, and nocturnal stridor. 

Sleep homeostasis is distinct from, but also linked to, the circadian clock. The illnesses most closely related to the circadian clock are the circadian rhythm sleep disorders, which include advanced sleep phase disorder and delayed sleep phase disorder [67]. Delayed sleep phase disorder is a sleep disorder in which there is a stable delay of the major sleep episode relative to the required sleep/wake clock time. Although delayed sleep phase disorder is common in adolescents and young adults, several reports have identified polymorphisms in the clock genes *Clock*, *Per3* and *CKIε* in patients with delayed sleep phase disorder [68,69,70]. Advanced sleep phase disorder is a sleep disorder in which there is a stable advance of the major sleep period, characterized by habitual and involuntary sleep onset and wake-up times that are several hours earlier than the desired or conventional clock times. Advanced sleep phase disorder is more common among middle-aged and older adults. A genetic basis has been clearly demonstrated in familial advanced sleep phase disorder, with missense mutations located in clock genes including *Per2*, *CKIδ* and *Cry2* [71,72,73]. Although there are no reports of miRNAs abnormally expressed in patients with circadian rhythm sleep disorders, abnormal changes in the expression and/or modification of clock genes by some factors, including miRNAs, could be a primary cause of these syndromes. 

Insomnia is a common clinical condition characterized by difficulty initiating or maintaining sleep, accompanied by symptoms such as irritability or fatigue during wakefulness, and is common in neurological diseases such as mood disorders, psychiatric diseases, prion diseases and neurodegenerative diseases [74]. Patients with circadian rhythm sleep disorder typically have insomnia, excessive daytime sleepiness, or both. However, there are relatively few clinical studies on patients with only insomnia, probably because insomnia itself is not lethal but rather is often reported as a symptom accompanying more serious diseases. On the other hand, it might be possible to predict future diseases if the causative miRNAs and clock genes common to patients with insomnia and patients with serious diseases could be identified. Interestingly, an SNP on the *Clock* gene has been associated with major depressive disorder, bipolar disorder and/or antidepressant treatment with sleep problems [75,76,77]. An SNP in miR-146a has also been associated with susceptibility to fatal familial insomnia, which is one of the prion diseases that interferes with sleep and leads to the deterioration of mental function and loss of coordination [78]. Genetic variants and abnormal processing of pre-miR-182 are present in major depression patients with late insomnia [79]. As described above, miR-182 could be a circadian modulator that shows circadian expression. These results suggest that aberrant circadian regulation and/or miRNA expression induce various diseases accompanied by insomnia.

Hypersomnia is characterized by either excessive daytime sleepiness or excessive time spent sleeping and includes both narcolepsy and idiopathic hypersomnia [80]. A genetic link between narcolepsy and the chromosomal region that contains the *Clock* gene has been established [81,82]. However, it is still unclear whether SNPs in *Clock* are directly associated with narcolepsy, since two of them have already been reported to have no relation to narcolepsy [83]. On the other hand, in idiopathic hypersomnia, the amplitude of the rhythmically expressed BMAL1, PER1 and PER2 is significantly dampened compared to that in healthy controls [84,85]. The levels of several miRNAs are significantly altered in the blood of narcolepsy patients, including miR-182-3p, which is the other strand of miR-182-5p regulating *Clock* expression [86]. Since a genetic variant in the precursor form of miR-182 causes major depression in patients with late insomnia, this miRNA may be involved in circadian and sleep function. Further, the aberrant miRNAs, miR-130a, miR-26a, miR-30c and let-7f, have been commonly detected in plasma from both patients with narcolepsy and patients with idiopathic hypersomnia [87]. There is a report showing that chicken miR-26a, which is highly conserved among *Drosophila*, chickens, mice and humans, shows diurnal rhythm through the regulation of CLOCK and CREB. This miR-26a regulates the protein level of the L-type voltage-gated calcium channel α1C subunit in chicken cone photoreceptors, implying that the regulation of light input by miRNA and clock genes is important for sleep regulation. 

Sleep-related breathing disorders, ranging from habitual snoring to increased upper airway resistance syndrome to sleep apnea, are now recognized as major health problems [88]. The majority of patients with sleep-related breathing disorders have excessive daytime sleepiness and tiredness. It has been reported that the expressions of the clock genes *Per1*, *Per3* and *Cry1* are deregulated in the patients with obstructive sleep apnea [89,90]. Further, several miRNAs were identified as deregulated miRNAs in patients with this symptom, and some of these miRNAs are associated with the expression of clock genes [91,92]. Among them, miR-181a is up-regulated in patients with obstructive apnea. MiR-181a plays a role in modulating circadian rhythm by targeting *Per3* in the stromal cells as well as *Cry1* in the kidney [51,93]. Furthermore, the downregulation of miR-27 and let-7 has been observed in patients with obstructive sleep apnea, and in silico data analysis suggest that these miRNAs could target the *Cry2* gene [94]. The up-regulation of miR-199b-5p is likely to be associated with obstructive sleep apnea, whereas its downregulation is involved in NPAS2 overexpression, leading to tumor cell survival through the reprogramming of glucose metabolism and reduction in oxidative phosphorylation [46]. In addition, miR-107, which is down-regulated in obstructive sleep apnea, is particularly abundant in the brain, and regulates the circadian system via targeting *Clock* in the epithelial cells [40]. The miRNAs that play a role in circadian rhythm in obstructive sleep apnea appear to be mostly related to cell survival, suggesting that sleep-related breathing disorders could be critical diseases because of the chronic abnormal expression of miRNAs and clock genes. 

Since sleep disorders are often observed as early symptoms of several diseases, chronic abnormalities in the function of circadian clock and its-related miRNAs may cause serious diseases. The identification of common abnormally expressed miRNAs related to circadian clock will assist in the early therapeutics of critical diseases before they become fatal.

## 7. Circadian Rhythm and Neurodegenerative Diseases

Neurodegenerative diseases are characterized by the progressive degeneration of the structure and function of the nervous system, and include Alzheimer’s disease (AD), Parkinson’s disease (PD), amyotrophic lateral sclerosis (ALS), Huntington’s disease (HD) and multiple system atrophy (MSA). These diseases primarily occur in the later stages of life. As an ironic side effect of the worldwide increases in life expectancy and resulting growth in the aging population, the number of people suffering from neurodegenerative diseases has also grown. Currently, no neurodegenerative disease is curable; the available treatments only manage the symptoms or halt the disease progression.

Circadian abnormalities have generally been considered consequences of neurodegeneration. Despite the varied pathogenesis and diversity of symptoms among the neurodegenerative diseases, it is common for patients with these disorders to exhibit disruptions in the circadian rhythms of physiological and behavioral processes, or circadian fluctuations in their symptoms. However, recent lines of evidence suggest that circadian disruption might actually contribute to the neurodegenerative process [95]. Several findings indicate that the circadian system may in fact play a more direct role in the etiology of neurodegenerative diseases [96]. 

Although there are several causes of the onset and progression of neurodegenerative diseases, oxidative stress enhancement is a common feature [97]. Oxidative stress has been defined as an imbalance of reductant and oxidant (Redox) states in which reactive oxygen species production exceeds the capacity of antioxidant systems to control it [98]. Interestingly, many of the genes involved in controlling the antioxidant system show circadian rhythm [99]. Moreover, BMAL1-deficient mice exhibit increased levels of reactive oxygen species (ROS) and accelerated ageing, suggesting that the circadian clock is involved in ROS regulation [100]. Although much emphasis has been placed on the role of protein aggregates in neurodegenerative diseases, a growing body of evidence is also converging on altered RNA processing as a contributing factor in the pathogenesis of neurodegenerative diseases [101]. MiRNAs are among the most important of the RNA-processing factors and play a post-transcriptional regulatory role [14]. The aberrant expression of miRNA is detected in the brain, cerebrospinal fluid and blood of patients with neurodegenerative diseases [101] (Table 1). 

### 7.1. Alzheimer’s Disease

AD is an irreversible age-related neurodegenerative disease characterized by progressive dementia developed in middle or later life. The pathological hallmarks are depositions of amyloid β (Aβ) plaques and neurofibrillary tangles composed of abnormally phosphorylated tau protein in the brain [102]. The ε4 allele of the apolipoprotein E gene (APOE) is a major genetic risk factor for late-onset AD. The ApoE4 protein enhances Aβ deposition in the CNS.

Common symptoms related to sleep in AD include difficulties in falling asleep, arousal at night, repeated awakenings and waking up too early in the morning, and sleepiness/frequent naps during the day. Obstructive sleep apnea also frequently occurs in patients with AD [103]. These symptoms are also evident in patients with mild cognitive impairment, suggesting that sleep disorders could be primary symptoms prior to the clinical diagnosis of AD. Nighttime sleep becomes increasingly fragmented as the disease progresses, while nocturnal activity levels and daytime sleepiness increase [104]. In severe cases, minimal differences exist between daytime and nighttime bouts of activity and sleep due to a flattening of the melatonin rhythm. Changes in the rhythm of cortisol release have also been observed. Further, a rhythm in mood and emotional volatility has been reported to emerge along with the progression of the neurodegenerative conditions. 

Aberrant BMAL1 methylation and transcription have been observed in the brains of AD patients, leading to alterations in BMAL1 expression and neuronal circadian rhythms, contributing in part to the sleep and behavior alterations associated with pathology [105]. Further, diurnal variations in PER1, PER2, and BMAL1 gene expressions are altered in several brain areas of AD patients [106]. Interestingly, Aβ itself shows a circadian pattern with an increased level during wakefulness and decreased level during sleep [107]. Decreases in Aβ circadian patterns with age and amyloid deposition have also been observed. Moreover, in a mouse model of AD, *Bmal1* regulated the expression of the APOE gene and *Bmal1* deletion caused a loss of Aβ rhythms in the hippocampus, resulting in marked increases in the amyloid plaque burden [108]. The physiological isoform of Aβ originates from the amyloid precursor protein via sequential cleavages that are catalyzed by BACE proteins (BACE1 and BACE2) and by presenilin proteins (PSEN1 and PSEN2) [109]. BACE1 is inhibited by the D-box repressor E4BP4, while BACE2 and PSEN2 are activated by CLOCK:BMAL1 [109,110]. Tau pathology in AD is related to tau phosphorylation and aggregation. One of the most important tau kinases is GSK3β, which is known to phosphorylate and modify the clock proteins, BMAL1, PER2 and CRY2 [111,112,113]. 

Although many miRNAs have been shown to be deregulated in the blood, cerebrospinal fluid and the brains of patients with AD, the lists of such miRNAs are not always consistent between studies, probably because of the small cohort sizes, circadian changes or discrepancies in the disease stage, underscoring the importance of systematic analysis from multiple clinical trials [114]. However, it is of interest to note that common miRNAs are involved in the expression of genes related to circadian rhythm, sleep disorders and AD pathogenesis, indicating the strong connection between them. The overexpression of miR-219 has been observed in the brains of patients with AD, and miR-219 is known to play a role in the downregulation of Tau phosphorylation by targeting GSK-3β [115]. This miRNA has been reported to be a modulator of the circadian clock via the CLOCK and BMAL1 complex [9]. MiR-132 is significantly downregulated in neurons in AD [116]. GSK-3β and Tau mRNA are targeted by miR-132 [117], which could be induced by photic entrainment cues via a MAPK/CREB-dependent mechanism [9]. On the other hand, miR-146a is up-regulated in several brain regions of AD patients [114]. The miR-146a is associated with short sleep [62,64,79] and has also been reported to show rhythmic expression [66]. This miRNA has also been suggested to be involved in the pathogenesis of AD by regulating the genes related to neuroinflammation and cerebrovascular dysfunction [118,119]. MiR-107, which is downregulated in the cortex of patients with AD, displays circadian rhythm [40]. Further, miR-107 is a candidate miRNA for participation in obstructive sleep apnea [91]. In addition, miR-107 could target *Clock* as well as BACE1, suggesting that miR-107 may trigger AD pathogenesis and its accompanying symptoms through the dysregulation of target genes [40,120,121]. MiR-26b is upregulated in AD, and MiR-26b has been identified as an oscillating miRNA in distinct high-throughput studies [22,23]. Interestingly, miR-26b has also been shown to contribute to Tau hyper-phosphorylation and Aβ accumulation [122]. Increased miR-34a expression was also observed in the temporal cortex region of patients with AD [123]. The rhythmic expression of miR-34a was observed in the tumor cell lines and its overexpression contributes to abnormal expression of the clock genes *Per1* and *Per2,* as well as the specific genes involved in memory formation, amyloid precursor protein (APP) metabolism and tau phosphorylation states [27,124,125]. In addition, hypoxia-induced miR-210 targets directly regulate various genes associated with the pathways of various diseases, including neurodegenerative disease [126]. The dysregulation of miR-210 in brain tissues, as well as in cerebrospinal fluid and serum, has also been correlated with the pathology of AD. MiR-210 is known to control circadian locomotor rhythms in *Drosophila*, and this effect might also be observed in mammals, since miR-210 was evolutionarily conserved between *Drosophila* and mammals [127,128]. Although miR-125b and miR-29b are the top-ranked AD biomarkers in several clinical studies [129,130,131], the mechanism of dysregulation in the blood of AD patients is still unclear. In silico analysis reveals that miR-125b regulates the cholinergic neuron functions by targeting *Clock* [132]. Further, recent evidence shows that miR-125b regulates neuronal cell growth and apoptosis via the regulation of inflammatory factors and oxidative stress, and this regulation may be related to AD pathogenesis [133]. On the other hand, miR-29 family members can target BACE1 mRNA and be downregulated in sporadic AD [134]. It is known that Period genes are regulated by the miR-29 family [43]. These results suggest that AD pathology, pathogenesis, and pathophysiology are strongly connected to circadian rhythm abnormalities, sleep disorders and miRNA dysregulation.

### 7.2. Parkinson’s Disease

PD is the second most common neurodegenerative disease after AD, and is clinically characterized by resting tremor, rigidity, akinesia, and postural instability [135]. PD is characterized as a progressive, late-onset movement disorder which is affected by dopaminergic neurodegeneration in the substantia nigra (SN). Lewy bodies, which are eosinophilic neuronal inclusions that contain both α-synuclein and ubiquitin, are pathological hallmarks of PD [136].

Since dopaminergic neurotransmission lies at the core of PD pathology, diurnal and circadian variation in dopamine content and metabolism should be considered when investigating the mechanism [137]. Several reports have reported diurnal variation in dopamine and some of its metabolites. Changes in dopamine content could be causally related to rhythms in dopamine-synthesizing enzymes and transporters, whose activities exhibit temporal changes [138]. Rhythmic dopaminergic activity can be controlled by the circadian clock and, in turn, might also regulate the activity of the clock itself [138,139]. In addition, dopamine might be relevant to the modulation of circadian retinal input [140].

Sleep disorders are among the most frequent non-motor symptoms of PD and include insomnia, parasomnia, nocturia, and sleep-related breathing disorder—all of these conditions lead to excessive daytime sleepiness, which usually increases in frequency over the course of the disease and disability progression [141]. Behavioral sleep disorders, such as restless leg syndrome and REM behavior disorder, are especially highly comorbid with PD [142]. Cortisol release is elevated in PD, although the diurnal pattern of cortisol remains rhythmic [143]. It has also been reported that BMAL1 expression is dampened in total leukocytes of patients with PD [144]. 

Clinical studies for PD patients show the dysregulation of several miRNAs in the whole blood, serum, or CSF. The miR-29 family members are particularly interesting in this regard, because the expressions of miR-29a and miR-29c are both down-regulated in PD [145]. Further, blood miR-29a is overexpressed in levodopa-treated patients with PD [146] and miR-29c overexpression attenuated dopaminergic neuron loss and α-synuclein accumulation in the SN of PD mice [147]. The targets of the miR-29 family also include PER proteins, although the rhythmicity of the miR-29 family has not been reported yet [21,148]. Since the expression of miR-29 family members is also altered in AD patients, the miR-29 family members are suggested to be key factors for neurodegeneration and clock gene dysregulation. In addition, several clinical studies have reported lower expression of miR-30c in PD patients [149]. MiR-30c has been reported to be elevated in patients with narcolepsy, and changes in the expression of MiR-30c in response to sleep deprivation have been observed in young patients with narcolepsy [87,150]. Although the actual targets of miR-30c have not been identified, a computational algorithm revealed many target genes that are involved in neuronal autophagy, mitophagy and the regulation of dopaminergic cell death [151]. In addition, circadian PER proteins are also predicted to be target genes of miR-30c [148], although the precise mechanisms by which miR-30c affects PD pathology and circadian rhythm are unknown. miR-19b levels have been reported to be lower in patients with PD compared to healthy controls, and the protective effect of miR-19b is mediated by targeting the genes related to neuronal apoptosis [152,153]. Further, the decreased expression of miR-19b has been observed in patients with idiopathic REM sleep behavior disorder several years before a diagnosis of PD or dementia with Lewy bodies [154]. MiR-19b could be a key regulator of the circadian transcripts CLOCK and RORα and has been shown to be influenced by estrogen and stress exposure [48]. The levels of miR-221 are also significantly decreased in PD patients compared with healthy control populations. MiR-221 has a protective role in PD by targeting a gene related to apoptosis and is modulated by DJ-1, whose loss-of-function mutations are linked to recessively inherited PD [155]. In addition, circadian oscillation of salivary miR-221 expression has been reported, suggesting that the circadian dysregulation of anti-inflammatory functions through miRNAs could be involved in PD pathogenesis [156]. Elevated levels of miR-126 may play a functional role in DA neurons and in PD pathogenesis by downregulating insulin signaling [157]. It has been reported that miR-126 is involved in the mechanism of ROS production [158]. Moreover, the altered expression of miR-126 is associated with insufficient sleep [64]. These facts indicate an interaction among PD pathogenesis, sleep disorders, oxidative stress and miRNA dysregulation.

### 7.3. Amyotrophic Lateral Sclerosis (ALS)

ALS is a chronic progressive disease characterized by selective degeneration of motor neurons in the spinal cord and motor cortex, normally causing death within 3–5 years of onset [159]. Cortisol rhythm is dysregulated in patients with ALS; in particular, evening cortisol levels are significantly increased in ALS patients compared to controls [160]. Sleep disorders, including nocturnal hypoventilation, restless leg syndrome, mood disorders, sleep-disordered breathing, and circadian disturbances, occur frequently in ALS patients [161]. 

Mutations in several genes have been identified as potential genetic risk factors for ALS. These include mutations in Cu/Zn superoxide dismutase 1 (SOD1), TAR DNA-binding protein 43 (TDP-43) and fused in sarcoma/translated in liposarcoma (FUS), and an increased number of repeats in chromosome 9 open reading frame 72 (C9orf72) [162]. The expression and activity of SOD1 show circadian variations, which are significantly dampened in *Per1*/*Per2* double-knockout (DKO) mice [163]. Further, TDP-43 regulates the circadian period by stabilizing CRY proteins [164]. Recently, FUS has been identified as a novel modulator for circadian gene expression positively regulated by REV-ERBα [165]. The hexanucleotide (GGGGCC) repeat expansion in the C9orf72 gene is the underlying genetic cause in approximately half of the familial amyotrophic lateral sclerosis (ALS) cases and in about 10% of the sporadic ALS cases [166]. Interestingly, aberrant protein aggregates in the SCN-related neuron of C9orf72-related ALS patients may disturb the circadian rhythm of their sleep/wake cycle [167]. 

Since it is evident that ALS affects not only neurons in the CNS, but also peripheral muscle tissues, circulating miRNAs may contribute to the association of central and peripheral organs in the ALS pathology. The most promising of the circulating miRNAs that are deregulated in ALS patients are miR-206 and miR-133a/b. Both these miRNAs are highly expressed in myocytes and up-regulated in the muscle and brain in patients with ALS [168]. Furthermore, other miRNAs, such as miR-142 and miR-132, have been reported to target a specific set of genes related to the pathophysiology of ALS [168]. 

The up-regulation of circulating miR-206 has been observed in ALS patients and is known to play a crucial role in the reinnervation process [169]. MiR-206 also has a profound effect on the dynamic mechanism of the mammalian circadian clock, both by control of the amplitude and the frequency to affect the level of the gene expression [170]. MiR-133a and miR-133b are involved in muscle proliferation, repair and regeneration, and both these miRNAs are up-regulated in ALS patients [171,172,173]. It has been reported that miR-133, which is highly conserved, contributes to core circadian gene expression based on the significant differences observed between a clock mutant and wild type fly [174]. In addition, miR-142 is up-regulated in the spinal cord of sporadic ALS patients [173]. MiR-142 has been predicted to target TDP-43 and C9orf72 [175] and is also known to regulate the expression of Nrf2, a transcription factor that controls the expression of antioxidant-response genes [176]. MiR-142 is one of the regulators of SIRT1, which has a deacetylase activity that counteracts the acetylase activity of CLOCK [177,178] and oxidative stress [179]. Moreover, the inhibition of Dicer function by ALS-causing mutant proteins, such as SOD1, TDP-43 and FUS, may lead to alterations in miRNA processing, which could account for some of the miRNAs whose expressions are altered in ALS [180]. MiR-132 has been reported to be upregulated in ALS patients [181]. Because miR-132 has been identified as a TDP-43-binding miRNA [175], it is directly affected by mutations in both TDP-43 and FUS in neuronal models of ALS [182,183]. Finally, it has been reported that miR-132 plays an important role for coupling the circadian clock to daily rhythms of neuronal plasticity and cognition [184]. These results indicate that ALS-causing genes are strongly correlated with circadian genes and miRNAs.

### 7.4. Huntington’s Disease

HD is caused by the expansion of *CAG* trinucleotide repeats (in excess of 38 repeats) on chromosome 4 in exon 1 of the gene coding “*huntingtin*” with autosomal-dominant inheritance [185]. HD patients show hyperkinetic movement disorders due to basal ganglion dysfunction. The most common sleep problems reported by HD patients are insomnia, difficulties in falling asleep, frequent nocturnal awakenings, and excessive daytime sleepiness [186].

Circadian gene expression is impaired in HD model flies [187]. Delayed acrophase of *per* and *tim* in HD flies correlates with delayed nighttime sleep. Impaired clock gene expressions of *Per2* and *Bmal1* are also observed in a mouse model of HD that shows disrupted night–day activity patterns mirroring the symptoms of HD patients [188].

Several miRNAs, such as miR-132, miR-124 and miR-146a, are involved in the association with HD [189]. Alterations of miR-132 expression are present in the brains of HD patients [190]. With respect to circadian physiology, miR-132 expression is under the control of the circadian oscillator in the SCN, and photic entrainment cues trigger a marked increase in the levels of miR-132 expression [9]. Altered expression of miR-132 is also observed in patients with AD and ALS, possibly because miR-132 is highly expressed in the brain and regulates multiple oxidative stress-related pathways [191]. It is of interest to note that miR-124 is one of the miRNAs down-regulated in the brain of HD patients [196] and miR-124 could slow the progression of Huntington’s disease by promoting neurogenesis in the mouse striatum [192]. MiR-124 is conserved across the animal kingdom and is abundantly expressed in the central nervous system. In flies, MiR-124 expression is under circadian regulation and modulates circadian output [193,194]. The expression of miR-146a shows circadian rhythm in human retinal endothelial cells and regulates the genes related to inflammatory response [66]. Further, miR-146a is upregulated in HD patients [195] and is known to target the HTT gene [196]. The genetic mutation of miR-146a causes fatal familial insomnia and a reduction in miR-146a expression is observed in people chronically short of sleep [64,78]. These results indicate that miRNAs are a key factor for the interplay of circadian abnormality, sleep disorder and Huntington’s disease.

### 7.5. Multiple System Atrophy

Multiple system atrophy (MSA) is a devastating neurodegenerative disease representing parkinsonism, cerebellar ataxia, autonomic dysfunction, and pyramidal signs [197]. MSA, clinically predominated by parkinsonism, is defined as MSA-P, while that predominated by cerebellar ataxia is called MSA-C. MSA patients show significant brain atrophy of the putamen, cerebellum, pons, or middle cerebellar peduncle with mild cortical atrophy in the frontal lobes. The mean age at the onset of symptoms is 55 to 60 years and the mean survival from the onset is 6 to 10 years. At present, there is no disease-modifying therapy and only symptomatic therapies, such as levodopa, are available for clinical use.

Sleep disorders in MSA are frequent and severe, and include insomnia, daytime sleepiness, restless legs syndrome, REM sleep behavior disorder, and sleep disordered breathing [198]. A postmortem study of the brains of patients with MSA revealed the degeneration of AVP neurons in the SCN [199], and such degeneration was subsequently shown to lead to the impairment of the circadian rhythm of plasma AVP concentration in MSA [200,201]. 

The number of clinical studies showing miRNA profiles in MSA patients is much lower than the numbers of such studies for other neurodegenerative diseases, simply because MSA is rarer. Several miRNAs are differentially expressed in the brain and body fluid of MSA patients compared to controls (e.g., miR-24, miR-96 and miR-433).

The level of miR-24 expression is dysregulated in the serum of patients with MSA [202,203]. Interestingly, miR-24 binds to the 3′-UTR of *Per2* and plays an important post-transcriptional regulatory role in the PER2 expression required for normal circadian timekeeping, as described above. Further, miR-24 is disrupted in the cohort of autistic children with disordered sleep patterns compared to those without sleep problem [156]. miR-96 has been identified as one of the up-regulated miRNAs in both MSA patients and a mouse model of MSA [204]. Further, miR-96 targets EAAC1, a transporter of cysteine, which is a precursor substrate of the antioxidant glutathione, as well as a taurine transporter. It has been shown that rhythmic miR-96 plays an important role in neuroprotection through its regulation of glutathione levels [205]. The expression of miR-433 has been reported to be downregulated in the cerebellum of post-mortem MSA cases [206]. Further, the down-regulation of miR-433 was also observed in the striatum of an MSA transgenic mouse model [207]. MiR-433 shows robust circadian rhythm and regulates *Per2* gene expression by regulating glucocorticoid receptors [208]. These findings indicate the importance of miRNA regulation in circadian rhythm and suggest that the disruption of miRNA regulation might play a role in the etiology of these neurodegenerative diseases.

## 8. Conclusions and Future Perspectives

**Figure 2:**
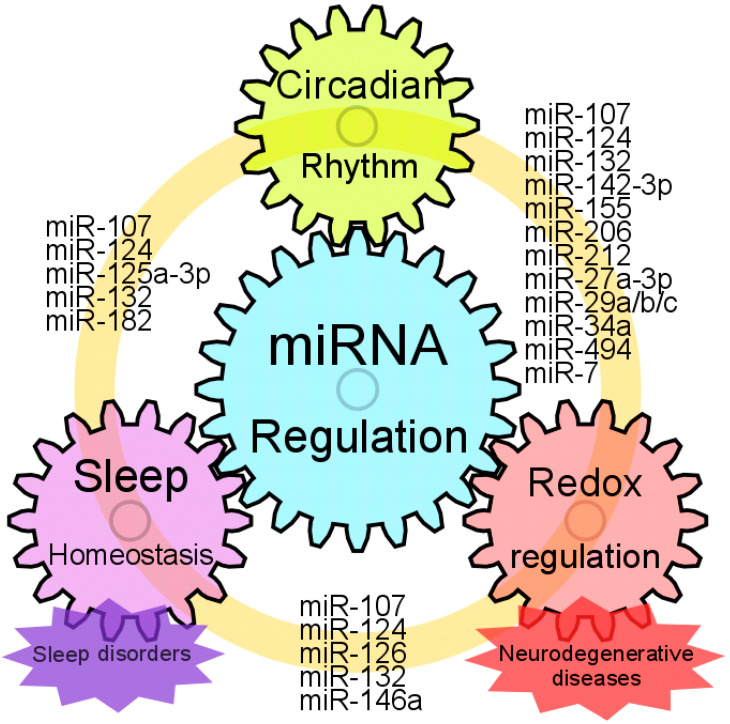
Models for interplay of miRNA regulation, circadian rhythm, sleep homeostasis and neuroprotection. Common miRNAs which could regulate circadian rhythm, sleep homeostasis and redox states are represented. Sleep homeostasis is strongly connected to circadian rhythm. Disruption of circadian rhythm causes sleep disorder, and vice versa. Patients with neurodegenerative disease often complain of sleep deprivation. Moreover, sleep disorders can be an early symptom of neurodegenerative diseases. Several miRNAs that are altered with the circadian abnormalities, sleep disorders or neurodegenerative diseases are dysregulated in the brain and blood of disease-model animals or patients with neurodegenerative diseases. Taken together, these facts suggest that miRNAs have the potential to be biomarkers as well as therapeutics for these diseases.

Sleep homeostasis is strongly connected to circadian rhythm, and abnormalities in both are often observed in patients with neurodegenerative diseases. Recently, emerging studies have suggested that sleep and circadian alterations precede neurodegenerative diseases and may contribute to disease progression. Growing bodies of evidence show that several circadian-related miRNAs are altered in both sleep disorders and neurodegenerative diseases, as reviewed in this study. These facts imply that the abnormalities in the expression of circadian miRNAs in patients with sleep disorders could be biomarkers for future development of neurodegenerative disorders (Figure 2). Moreover, manipulation of the expression of miRNAs in the early stage of diseases could be used as a treatment for sleep disorders as well as neurodegenerative diseases. Further research is needed to develop therapeutics for neurodegenerative diseases that are currently incurable and progressive.

## Figures and Tables

**Table 1:** Dysregulated miRNAs in the patient with neurodegenerative diseases, which is related to the regulation of clock genes or sleep disorders.

Disease	miRNA	Target Clock Gene	Rhythmicity	Regulation	Predicted Disease Mechanism	Related Sleep Disorder	Reference
Alzheimer’s disease	miR-107	Clock	rhythmic	**temporal cortex** ** ** **↓**	Increased BACE1 expression	obstructive sleep apnea	[40,91,114,120,121]
				whole blood ↓			
				plasma ↓			
	miR-125b	Clock	n.d.	**hippocampus** **↑**	Increased BACE1, APP and Tau protein expression	n.d.	[114,129,130]
				serum ↓			
	miR-132	n.d.	rhythmic	**temporal cortex** ** ** **↓**	Pathological aggregation of tau protein	n.d.	[9,116,117]
				serum ↑			
				exsome ↓			
	miR-146a	n.d.	rhythmic	**frontal cortex** **↑**	Increased tau hyperphosphorylation	chronic short sleep	[62,64,66,79,114,118,119]
				**hippocampus** **↑**		fatal familial insomnia	
				plasma ↓			
				serum ↓			
	miR-210	Per (Drosophila)	n.d.	**hippocampus** **↓**	Dysregulation of hypoxic stress pathway	n.d.	[114,126]
				CSF ↓			
				serum ↓			
				plasma ↑			
	miR-219	n.d.	rhythmic	**entorhinal cortex** **↓**	Accumulation of insoluble tau	n.d.	[115]
	miR-26b	n.d.	rhythmic	**temporal cortex** ** ** **↓**	Induced tau hyperphosphorylation	n.d.	[22,23,114,122]
				whole blood ↓	Aβ accumulation		
				serum ↓			
	miR-29a/b	Per1	rhythmic	**cortex** **↓**	Increased BACE1 expression		[129,130,131,134]
		Per2	(primary transcript)	whole blood ↓		n.d.	
		Per3		serum ↓			
				plasma exosome ↓			
				blood mononuclear cells ↓			
	miR-34a	Per1	rhythmic	**temporal cortex** **↓**	Accumulation of intraneuronal Aβ	n.d.	[27,114,124,125,129,130,131]
		Per2		**hippocampus** **↑**	Induced tau hyperphosphorylation		
				**frontal cortex** **↑**			
				plasma ↓			
				blood mononuclear cells ↑			
Parkinson’s disease	miR-126	Dbp	n.d.	**dopaminergic neurons** **↑**	Dysregulation of trophic support in DA neurons	chronic short sleep	[64,149,157,158]
				blood mononuclear cells ↓			
	miR-19b	Clock	n.d.	**CSF** **↓**	Promotion of cell apoptosis	idiopathic REM sleep	[48,149,152,153,154]
		Rora		serum ↓		behavior syndrome	
				blood mononuclear cells ↓			
	miR-221	n.d.	rhythmic	serum ↓	Inhibition of cell proliferation	n.d.	[155,156]
					Promotion of apoptosis		
	miR-29a/c	Per1	rhythmic	serum ↓	Doperminergic neuron loss	n.d.	[21,145,146,147,148,149]
		Per2	(primary transcript)	blood mononuclear cells ↓	α-synuclein accumulation		
		Per3					
	miR-30c	n.d.	n.d.	serum ↓	Progression of α-synucleinopathies?	narcolepsy	[148,149,151]
				blood mononuclear cells ↓	(Predicted by computational analysis of gene network)		
Amyotrophic lateral sclerosis	miR-132	n.d.	rhythmic	muscle ↑	Inhibition of neurite outgrowth	n.d.	[175,181,182,183,184]
	miR-133a/b	n.d.	n.d.	**spinal cord** **↓**	Involved in muscle proliferation and regeneration	n.d.	[171,172,173,174]
				serum ↑			
	miR-142	Bmal1	rhythmic	**spinal cord** **↑**	Promotion of ALS pathogenesis	n.d.	[173,175,176,177,178]
				serum ↑			
	miR-206	Clock	rhythmic	serum ↑	Involved in reinnervation process	n.d.	[169,170]
Huntington’s disease	miR-124	Clock	n.d.	leukocytes ↓	Disease progression	n.d.	[189,192,193,194]
	miR-132	n.d.	rhythmic	**frontal cortex** **↓**	Enhancement of oxidative stress	n.d.	[9,189,190]
	miR-146a	n.d.	rhythmic	**frontal cortex** **↑**	Targeting Huntingtin gene	chronic short sleep	[64,66,78,195,196]
				**striatum** **↑**		fatal familial insomnia	
Multiple system atrophy	miR-24	Per2	rhythmic	**CSF** **↓**	Involved in cerebellar degeneration	disordered sleep patterns	[156,202,203]
				serum ↓		(autistic children)	
				plasma ↓			
	miR-433	Per2	rhythmic	**cerebellum** **↓**	Involved in formation of glial cytoplasmic inclusions	n.d.	[206,207,208]
	miR-96	n.d.	rhythmic	**frontal cortex** **↑**	Inhibition of transporters involved in antioxidant defense	n.d.	[204,205]

Dysregulated miRNAs in patients with neurodegenerative diseases, which are related to the regulation of clock genes or sleep disorders, are listed. Listed here are miRNAs mentioned in this review, although there are many other dysregulated miRNAs identified as a predicted biomarkers and/or therapeutics. CSF, cerebrospinal fluid; n.d.; not determined; Bold font, miRNA regulation in the brain tissue or CSF.
